# Resources, Problems and Challenges of Autism Spectrum Disorder Diagnosis and Support System in Poland

**DOI:** 10.1007/s10803-021-05142-1

**Published:** 2021-08-03

**Authors:** Anna Lenart, Jacek Pasternak

**Affiliations:** College of Social Sciences, Institute of Pedagogy, Department of Psychology, University of Rzeszow, ul. Ks. Jalowego 24, 35-010 Rzeszow, Poland

**Keywords:** Autism spectrum disorder, Diagnosis, Comorbidity, Overshadowing

## Abstract

The article refers to resources, problems and challenges of autism diagnosis and support system in Poland. The resources include: the increasing number of specialists, diagnostic and therapeutic centres, well-established course of education for people working with youths, standardised and normalised diagnostic tools. The diagnostic process is not without some areas in need of our focus: the tendency of some specialists to make unauthorised diagnosis, overshadowing; underestimation of comorbidity of ASD with other disorders. The challenges refer to introducing an effective system of monitoring the services provided in form of certification and control in order to prevent their abuse, initiating category of temporary diagnosis; paying more attention on individual’s resources, better cooperation among specialists, teachers and families, developing and unifying diagnostic standards.

An important part of the psychologist’s work is the process of discerning an individual’s strengths, observing the pathomechanism behind the formation of their disorder and looking closely at psychopathological symptoms. Yet some psychologists, clinicians and therapists feel that the term “diagnosis” itself has negative connotations, as they have witnessed situations in which psychological diagnosis has been wrongly applied. This occurs when, for example, a complex personality is reduced to a caricature by a therapist or diagnostician who struggles to cope with equivocality (McWilliams, 2009). According to Klajs (2017), making a diagnosis seems to be an essential condition for the overall operation of the healthcare system as it enables specialists to obtain remuneration for their work from an intermediary institution. Without a diagnosis there is no disease, without a disease there is no treatment, without treatment there is no payment. Developmental disorders should be recognized in the context of the family and specific situations and labelling them as norms or pathologies should be avoided as far as is possible. Klajs actually thinks that nosological units in psychiatry are theoretical constructs, arbitrarily adopted by a selected group of specialists and divided into categories and subcategories which make up classifications that change from time to time. This is exactly the situation we have today while we await the latest version of the ICD classification in force in Europe, including of course Poland, with version 10 to be replaced by version 11, published in 2018 (WHO (2018)—*World Health Organisation*), which will become effective in January 2022 (www.who.int).

The aim of this article is to present the diagnostic process of autism spectrum disorder (ASD) in Poland, its resources, and the risks and challenges faced by professionals involved in diagnosis and treatment.

Autism has been present in the Polish academic debate since 1980s but it is the SYNAPSIS Foundation, founded in 1989–1990, that has made a lot of progress support provision to people with ASD. This gave grounds to the development of research and the spreading of knowledge about ASD in Poland as well as to many initiatives which helped expand the support system.

There have been no official epidemiological studies on the prevalence of ASD in Polish population. Such attempts are made, but the results are limited to a particular region of Poland. Recent studies from two Polish regions indicate the prevalence at the level of 32–38 per 10,000 (Skonieczna et al., 2017). Data collected within the ASDEU project (*Autism Spectrum Disorders in the European Union*) indicate an average prevalence of 12.2 per 1000 (1:89) children aged 7–9 years. In European countries, it is estimated at 4.4–19.7 (Posada de la Paz, 2018). US data indicate that Autism Spectrum Disorders can affect even 1 in 54 children and the rate is higher than previous estimates from 2014 (Maenner et al., 2020; Baio et al., 2018). The increasing prevalence and incidence of ASD is also visible in Poland (Piskorz-Ogórek et al., 2015). The same disorder affects 1–2% of the population, making the problem of supporting people with autism spectrum disorders and their families an important social and research issue. Autism is a multifactorial neurodevelopmental disorder. Genetic variants classified as aetiological factors were identified in one study in only approximately 25–35% of ASD patients (Wiśniowiecka-Kowalnik & Nowakowska, 2019). The etiology of the disorder is not fully understood. Its diagnosis is usually based on detailed observation of the severity of symptoms and behaviours characteristic of the disorder, supplemented by an interview with the caregiver or the diagnosed person herself (in case of adults). According to the International Classification of Diseases ICD-10, autism belongs to the group of pervasive developmental disorders (F.84) (WHO, 2009). Its characteristic symptoms, the so-called autistic triad, include deficits in social communication and repetitive, restricted patterns of behaviours, interest and activity. Another classification used worldwide is the DSM (Diagnostic and Statistical Manual). According to its latest version of 2013, DSM-5 symptoms characteristic of autism are observed in two main areas: deficits in social communication and restrictive, repetitive patterns of behaviour (APA, 2013). A meta-analysis of studies on this topic has shown that the number of people whose ASD diagnosis has not been maintained according to the DSM-5 criteria is in the range of 25–68% (Kulage et al., 2014).

## Diagnostic ASD Procedures Available in Poland

The specificity of the disorder imposes a particular diagnostic procedure, based largely on the observation of symptoms and behavioural criteria, which raises questions about the reliability and accuracy of diagnoses, especially in young children (Pisula, 2013) or elderly people (Baron-Cohen et al., 2005). There are many combinations and configurations of symptoms leading to this diagnosis. With regard to the DSM-IV criteria, their number is 2027, while the new version of DSM-5 is limited to 11 possible combinations of criteria leading to an ASD diagnosis (McPartland et al., 2012). This means that as much as it is possible researchers continually try to objectivize the diagnosis and create diagnostic tools that would provide for and facilitate accurate diagnosis, as well as differentiate ASD from other disorders or diseases. The field related to the search for early symptoms of this disorder is developing particularly strongly at present (cf. Larsen et al., 2018; Pisula, 2013), as is the creation of tools that are sensitive enough to diagnose young children. Early diagnosis allows for a quick start of the therapy, thus increasing the patient’s developmental chances and reducing future funding required to help people with ASD (Chojnicka & Płoski, 2012).

In Poland, the quality and reliability of diagnosis depends largely on how widespread the ASD screening is, the adaptations diagnostic tools, and the training process of professionals-diagnosticians in the use of internationally established diagnostic tools as well as their ability to differentiate between the various disorders co-occurring with ASD. These issues are presented further in the article in reference to the Polish and world literature, and also to the authors’ own clinical experience as psychologists. The need for the fine tuning of the process of choosing appropriate therapy for a person with ASD and create an effective system of life-long support is also mentioned.

Screening is an important part of the early detection of developmental disorders, usually involving a large group of children (e.g. under 3 years of age) or targeting a group with a high risk of developmental disorders (e.g. children born prematurely or those with siblings with ASD). Of course, screening does not replace a comprehensive, in-depth diagnosis, but it does help to identify children who might need support in their development in order to plan their educational pathway or monitor development. An example of the first kind of initiative is the Polish the Early Identification of Autism Programme ‘Badabada’ which allows parents to check their child’s development through a website and M-CHAT-R/F tool (Robins et al., 2014). This is an internationally known questionnaire for carers that enables them to assess the risk of disorders from the autism spectrum. However, it is still not a programme that can be defined as nationwide as its coverage is insufficient but it should be stressed that intensive efforts are being made to make this happen (http://badabada.pl/o-nas/model-badan-przesiewowych). There are also other screening tools available in Poland for developmental disorders, such as Q-CHAT (Quantitative Checklist for Autism in Toddlers, Allison et al., 2008) for children aged 18–24 months, AQ-Child (The Autism Spectrum Quotient, Children’s Version, Auyeung et al. (2008), Polish version by Pisula et al. (2010); Rynkiewicz and Łucka (2018) for children aged 4 and over; ESAT (Early Screening of Autistic Traits Questionnaire, Dietz et al., 2006) for children from 14 months, STAT (Screening Test for Autism in 2-Year Olds, Stone et al., 2000) for children aged 24–36 months. A detailed description of these tools can be found in: (Pisula, 2013; Chojnicka & Płoski, 2012).

Due to the specific character of the manifestation of the autism symptoms, a comprehensive specialist diagnosis, which is another element of the process, should be carried out by a team of different specialists, in keeping with current diagnostic standards and using dedicated diagnostic tools. This is important particularly because the diagnosis of ASD is largely based on the observation of the child and a detailed interview with the caregivers, i.e. using information that requires knowledge and experience in their correct—i.e. compliant with the applicable diagnostic criteria—interpretation. Notably, in Poland the person authorised to make the final diagnosis is a medical doctor, usually a psychiatrist. Unfortunately, there is a tendency among other specialists, e.g. teachers, psychologists, and educators, to make unauthorised diagnoses or suggest them to the parents, which—without a detailed and often time-consuming diagnostic procedure—is quite irresponsible, ethically questionable, and often harmful. Ochojska and Pasternak (2020) have discussed this issue in detail, in the form of case studies and possible interpretations of this phenomenon. The analysis of interviews with caregivers of children referred for ASD assessment shows that many parents say that it was the teacher in the nursery or at school who recommended the diagnosis. In fact, teachers are sometimes the first people to use the term ‘autism’ in relation to children. This is particularly important in the context of research conducted among teachers regarding knowledge about ASD which, although regularly expanding, is still not free from oversimplifications, ignorance of the basic symptoms of autism or its prevalence in the population (Nowakowska & Pisula, 2018). This also applies to issues related to differentiating between mental disorders.

The most popular and used worldwidely instruments in the diagnosis of autism spectrum disorders in the USA, Australia and Western Europe are ADOS (Autism Diagnostic Observation Schedule, Lord et al., 2000) and ADI-R (Autism Diagnostic Interview-Revised) defined as the gold standard in ASD diagnosis. Their application realizes the postulate of standardization and allows for the unification of the results of the diagnosis and elimination of extremely varied diagnoses by specialists of the same field. ADOS has Polish standardization and normalization, whereas according to the information obtained from the Psychological Test Laboratory of the Polish Psychological Association (PTP), ADI-R is in the process of being made available to clinicians in Poland. ADOS-2, combined with a comprehensive ADI-R interview, helps us gather comprehensive information on the development of the person diagnosed, providing the basis for the assessment of the abnormalities observed which are characteristic for autism spectrum disorders. ADOS-2 (Autism Diagnostic Observation Schedule, Second Edition, Chojnicka & Pisula, 2017) is a standardized and semi-structured observation protocol. This version has been used effectively since 2012 and the Polish version has been available since 2017. In Poland, the use of the tool in both clinical and research practice requires training and only certified specialists are allowed to purchase it and apply in their research. The fact the training accessibility in Poland is limited to a few specialists-trainers, the tool is costly and relatively new contributes to the situation in which only few diagnosticians take advantage of the tool.

Certification is not, however, the mark of the level of competence (Rynkiewicz et al., 2018), and the method alone is not enough to make a reliable diagnosis. More information about the tool can be found in the papers by Chojnicka and Pisula (2017), Chojnicka and Płoski (2012). The second ‘gold standard’ tool is ADI-R (Lord & Rutter, 1994). In Poland, it is currently only approved for use in scientific research (Chojnicka & Płoski, 2012) as the validation process is still underway.

It is also worth mentioning that as of recently we also have a standardized polish version of the popular Psychoeducational Profile, Third Edition—PEP-3-PL tool (Pisula, 2019) used to measure the level of development of young children with developmental disorders, especially from the autism spectrum, in the trend of functional diagnosis. The tool also helps diagnose children with serious developmental delays or intellectual disability, which often coexist with autism (Pisula, 2012). Importantly, the current version of the tool has been validated in the Polish population of both typically developing children and those with ASD.

The Polish version of the ASRS (Autism Spectrum Rating Scales, Goldstein, Naglieri 2010—Polish version Wrocławska-Warchala & Wujcik, 2016) tool is also available, allowing for the diagnosis of children aged 2–18 years. The tool has several versions depending on the user (parent, teacher) and the age of the child. Full and short versions are available, the latter can be used in screening. The tool can also be helpful in the evaluation of the effectiveness of therapeutic programs. It can be used by psychologists and other professionals yet training in the application of the tool and interpretation of results is recommended or even mandatory for specialists other than psychologists.

The tools and procedures discussed so far in relation to the diagnostic process prevailing in Poland are important steps towards the provision of reliable professional diagnosis in this population of children, especially given the nature of their difficulties and the disorders coexisting with ASD, such as intellectual disabilities which require the diagnostician to analyse and verify additional information that may affect the final outcome.

## Comorbidity and Differentiating ASD from Other Disorders

The diagnosis of comorbidities is closely related to differential diagnosis. One should always consider whether the child has two separate disorders, one of which is ASD, or whether he or she needs to be diagnosed with a single disorder. Diagnosticians should ponder over different perspectives, for example whether poor eye contact and little social initiative are evidence of ASD or whether the child is perhaps depressed or socially phobic. Whether the child is autistic, introverted, or even schizoid, or perhaps punished by demanding parents, or even abused or overshadowed by older and more expansive siblings—these are examples of useful questions that must be part of diagnostic considerations. The following are various tips and differentiating factors that are worth reflecting in a differential diagnosis. ASD often coexist with other mental disorders (McCauley et al., 2020; Rybakowski et al., 2014), however, the authors feel that this is insufficiently reflected in the diagnostic documentation of many Polish children, which is often too one-sidedly focused on autism. These are also the conclusions reached by the authors of this paper, following the analysis they carried out of the diagnostic documentation of children with ASD. Similar observations have been made by specialists from other diagnostic centres, as reflected in the papers given at various conferences regarding the ASD problem areas. Although differential diagnosis is difficult, many professionals believe that ASD treatment alone will not lead to improvements in other emotional and behavioural spheres, hence the need for such diagnosis and specialist interventions. It is also common to attribute symptoms of other disorders to the primary diagnosis of ASD, which is known as *overshadowing* (Deprey & Ozonoff, 2017; Kerns et al., 2015). Diagnostically, the dichotomy ASD versus non ASD is too often considered which excessively simplifies the complexity and diversity of an individual’s mental functioning. In addition, closer to mental norm standards of behaviour or systemic and environmental behavioural contexts are quite often verified from the theoretical and conceptual perspective of ASD. The sense of otherness, isolation, misunderstanding, insufficient support, or gratification from the social environment by individuals with ASD may be one of the important reasons for appearing of other disorders, such as anxiety or depression. It therefore seems relevant to ask how to assess the patients’ self-esteem and level of satisfaction with life and how to help them attract attention, positive reinforcement and a satisfying quality of life—and also how to help them avoid negative experiences such as bullying and violence (Posar & Visconti, 2019). This perspective seems to be important in terms of our knowledge about comorbidity and differentiation of disorders in Poland, hence more space is devoted to these issues in this work.

It is estimated that about 70% of children with ASD develop other emotional or behavioural disorders, and that 40% will develop more than one (Levy et al., 2010; Leyfer et al., 2006). A recent meta-analysis of studies indicates a trend towards an increase in the number of diagnosed disorders associated with ASD (Rubenstein et al., 2018). It is then significant to distinguish whether the occurrence of symptoms such as anxiety is due to ASD itself, or whether it has been intensified for a specific period of time or occurs in connection with some particular situations in the individual’s life. A meta-analysis of research conducted over the last quarter of a century indicates that ASD is most commonly associated with ADHD (28%), anxiety disorders (20%), sleep–wake disorders (13%), disruptive, impulse control, conduct disorders (2%), depressive disorders (11%), obsessive–compulsive disorder (9%), bipolar disorders (5%) and schizophrenia spectrum disorders (4%) (Lai et al., 2019). According to Deprey and Ozonoff (2017), mood disorders, anxiety disorders, ADHD, tics, and psychotic disorders are the most common comorbidities occurring with ASD. The above meta-analysis did not include intellectual disabilities. It should be noted that the diagnosis of a child with ASD and co-occurring intellectual deficits is a challenge for several reasons. One of the most critical is linked to the limitations resulting from personal intellectual difficulties and the ability to take tests in a standard procedure, but there is also the limited number of tools which consider the specific nature of the functioning of people with ASD, but also with other co-occurring impairments (Rybakowski et al., 2014; Thurm et al., 2019). The American Centre for Disease Control and Prevention (CDC) indicated in 2014 that the prevalence of Intellectual Disability (ID) among children with ASD aged 4 years was estimated at 46% (Christensen et al., 2019), although Dykens and Lense (2012) in their meta-analysis indicated 65% of people with ASD with accompanying intellectual disability (ID). As far as cognitive abilities are concerned, 24 studies found that 32% of respondents with ASD reached average IQ scores (after: Rybakowski et al., 2014). Whereas earlier studies estimated that a reduced level of intellectual development of people with ASD concerned 40% to even 88% of this population (Fombonne, 2009), a decrease is currently being observed, most probably due to the extension of diagnostic criteria (Yates & Couteur, 2012; Wing & Potter, 2017). Studies conducted since the 1980s have shown that levels of cognitive abilities and linguistic development were one of the best predictors of the future development and functioning of these children (Brignell et al., 2018; Howlin et al., 2013; Posar & Visconti, 2019). The concern here is whether the results, especially for younger children with ASD, correspond to their real abilities, given the often poor understanding involved, especially in relation to verbal communication or social withdrawal. Research shows that with the appropriate choice of methods (e.g. non-verbal), it is possible to work with the child and obtain reliable results, with IQ (intelligence quotient) results not differing significantly in relation to previous IQ test results (Howlin et al., 2004). This shows the importance of this type of diagnosis being made by experienced diagnosticians for prognostic purposes, but it also calls for caution when it comes to the formulation of the diagnosis, especially in younger preschool children, as several studies have shown a later improvement in their general IQ scores (Klinger et al., 2017). No tool currently used in Poland for the assessment of intellectual ability has separate norms for diagnosing the intellectual abilities of people with ASD, especially considering their difficulties with speech and communication that are typical for this disorder. However, attempts are made to study these clinical populations in the process of the validation and standardization of tests and their inclusion in tool manuals, which is a valuable guide for diagnosticians. In Poland, such data are included in the IDS Intelligence and Development Scales (Jaworowska et al., 2012) and the latest version of the Leiter International Performance Scale—Third Edition, Leiter-3 (Jaworowska & Matczak, 2019). It should also be kept in mind that many of these children present an unharmonious cognitive skills profile, and even with recognized intellectual disabilities they may present strengths in particular fields or areas, such as memory. It is hard to differentiate between ASD and intellectual disability (ID) in young children, especially those who have not yet developed language or symbolic functions (they also exhibit repetitive behaviour). The differential diagnosis should focus on considering the importance of social communication disorders; if they are disproportionate higher to intellectual development, both diagnoses should be considered, if not just ID. In addition, significantly limited interest and repetitive behaviours indicate the presence of ASD (Pedersen et al., 2017). Motor stereotypies are part of the diagnostic criteria for ASD, so they usually do not require separate diagnosis. However, when stereotypic movement disorder cause self-harm and become a target of separate therapeutic interventions, both diagnoses are approved (APA, 2013).

Some of the disorders may manifest themselves differently in the course of ASD. Thus, 10% of people with ASD have been demonstrated to experience severe depression and 24% subclinical depression (Leyfer et al., 2006). The difference in the manifestation of depressive symptoms may be that the patient might not be feeling guilt but might display new or intensified aggression, agitation, self-harming behaviour, compulsive behaviour, loss of interest in particular topics, loss of control over defecation, reduced activity, reduced functioning in different areas, and difficulties with describing their lowered mood. The presence of comorbid disorders can be captured in terms of changes in relation to the past which are understood as psychopathological (e.g. less time spent talking about special interests may indicate anhedonia). The effect of such change may manifest itself in a lower frequency or extent of adaptive behaviour and deterioration of functioning, as well as the occurrence of additional problems that are not included in the ASD diagnostic criteria (Deprey & Ozonoff, 2017, pp. 357–360). The presence of mood and sleep disorders, in addition to the severity of the ASD symptoms and the marital status of the primary caregiver, are the main, though independent, factors that increase the risk of hospitalization in children with ASD (Righi et al., 2018). The likelihood of suicidal thoughts or behaviours in people with ASD is higher than in the general population (11–66%). The risk of attempted suicide may be higher in victims of bullying, rejection, tension related to social camouflage, many adverse or traumatic events, unmet needs for social support, and the consequence of comorbid psychiatric disorders additional to those in unfavourable course of ASD. The majority of research suggests that this is more often the case for people with HFA (high-functioning autism) or Asperger syndrome (Paquette-Smith et al., 2014; Weiner et al., 2019).

Comorbidity of anxiety disorders in children with ASD of pre-school age corresponds to the population average and increases in school age and adolescence. Children with PDD-NOS and Asperger’s syndrome have higher level of anxiety than children with autism at school age (Weisbrot et al., 2005). About 40% of children with ASD are affected by anxiety disorders (van Steensel et al., 2011). The most common are specific phobias, especially fear of thunderstorms, darkness, animal-related phobias, use of medical services and situational phobias (Deprey & Ozonoff, 2017). Anxiety in social situations is characteristic, without the fear of negative judgement, excessive fear of changes, new situations, increased worrying related to specific interests, as well as various types of phobias, e.g. concerning hand dryers (Kerns et al., 2014). Increased anxiety has a negative impact on an individual’s functioning: it escalates the symptoms of ASD, increases internal tension, irritability, anger, self-harm, suicidal ideation, makes it difficult to treat ASD; it also raises parental stress and anxiety, making it difficult for the patient to establish social relationships gain independence (Drahota et al., 2011; Kerns et al., 2014, 2015). When distinguishing between anxiety in ASD and Anxiety Disorders (AD), it has been noted that people with ASD experiencing social anxiety show little or no interest in social contact. On the other hand, people with AD experience anxiety about social contact due to the anticipated negative judgment by others; repetition of behaviour plays a role of pleasant gratification—soothing in ASD and reducing tension or compulsion in AD. Also, whereas perseverations are expressed in the form of repetitive speech in autism, they are the expression of real fears producing negative scenarios in anxiety disorders. Anxiety disorders can be a consequence of cognitive impairment and the resulting difficulties in understanding certain phenomena, such as a lightning occurring during a storm (Lainhart, 1999), whereas the level of anxiety is more constant, resembling anxiety as a trait (Leyfer et al., 2006). When a shy child with high levels of anxiety follows a pattern of withdrawal from social interactions or their avoidance, she or he does not learn to establish relationships, becomes socially clumsy and eventually stops trying to share his or her interests and feelings with others. It is therefore important to see whether the child has developed compassion, empathy, whether they can “be funny”, have a realistic view of themselves and whether they have developed an adequate theory of mind (ToM). If the answer to these is positive, ASD should be excluded (Gensler, 2012). Some studies have demonstrated that social anxiety is higher among primary school pupils attending public schools, compared to those who are in special schools (according to their carers), which may indicate greater emotional responsiveness in a more complex social environment, or may be associated with an increased social awareness among better-performing children with ASD (Zainal & Magiati, 2019). ASD can be confused with OCD because of the need for sameness, cognitive rigidity, a search for sensory stimuli and ritualistic behaviours. An important differentiating factor is that patients with OCD clearly experience anxiety when the ritual is not completed, while to those with ASD rituals seem to be enjoyable and are more ego dystonic. In ASD, touching or tapping objects is more of a search for stimuli, represents self-stimulation rather than compulsion, and is not intended to minimize anxiety quickly or counteract intrusive thoughts (OCD).

ADHD diagnosis in the course of ASD is associated with certain pragmatic consequences. ADHD may adversely affect the patient’s functioning, and specific methods of autism therapy might only have a negligible effect on the symptoms of ADHD. Typical features of ADHD in ASD include: difficulty in listening and following commands, maintaining order, sitting motionlessly, waiting for one’s turn, and excessive talking and interrupting others; these symptoms make it difficult to distinguish between the two disorders because they can be also traced back to ASD (Deprey & Ozonoff, 2017). Notably, that attention deficit problems in the context of ASD are qualitatively different from those in ADHD; excessive focus and internal distraction are characteristic of ASD, and insufficient focus and distraction from external events are characteristic of ADHD (Hendren, 2003). Hyperactivity decreases with age, often leaving only inattention and distraction in adulthood (Tantam, 2003). Social function disturbance associated with overactivity or impulsiveness and consequent rejection by peers should be distinguished from social disengagement, isolation and indifference to non-verbal aspects of communication in ASD (facial expression, voice tone). Research by Rynkiewicz and Łucka (2018) showed that 20% of women and 47% of men with ASD displayed ADHD symptoms prior to final ASD diagnosis.

The diagnosis of ASD in association with other known medical or genetic conditions (e.g., ASD associated with Rett syndrome, fragile X syndrome, Down syndrome, epilepsy, history of environmental exposure to a harmful agent or FAS, very low birth weight) may provide additional information. For example, it is estimated that 70–80% of boys with Fragile X syndrome (FXS), which is also the leading cause of intellectual disability, exhibit symptoms of autism (Will et al., 2019).

The decline in the development of social interaction can be seen in the regressive phase of Rett syndrome (1–4 years), which may resemble ASD symptoms. However, this is followed by a marked improvement in communication skills (APA, 2013). Rett syndrome (still present in ICD-10) was excluded from the spectrum as its genetic background has been identified (Volkmar & Reichow, 2013). In sensory processing disorder, ASD-like symptoms appear as a defence against undue stimulation. These include withdrawal, excessive attunement to stimuli, rigid or obsessive behaviour to feel more in control, and resistance when comfortable routines are threatened. Not all people with sensory processing disorder exhibit symptoms of ASD, and it is not clear whether all people with autism have sensory integration problems (Gensler, 2012). Emotional neglect or psychosocial deprivation with language delay, limited interests and low social skills should also be mentioned in this context. Such children are different from ASD in terms of improved social reciprocity and the likelihood of their achieving significant improvement after a therapeutic intervention. Consideration should also be given to individual adaptation problems that may temporarily be similar to ASD symptoms (Ochojska & Pasternak, 2020; Suthar et al., 2020)**.**

## ASD Therapy in Poland

The next step in the life of the family with a child diagnosed with ASD is the implementation of treatment and therapy, so it is worth to consider some of the therapeutic practices used in Poland in comparison with international standards and discuss the existence of various uncertainties about how to treat and support people with ASD. In spite of the large database of information on the subject, access to it is still insufficient. This applies to specialists, and also those working in specialist centres (Waligórska et al., 2012), which creates a considerable scope for promoting or applying interventions that do not objectively improve the functioning of children with ASD, but are attractive in form and promise quick results, which often proves to be encouraging to parents. To counteract these practices and raise awareness among professionals who support people with ASD worldwide, reports such as those from the National Autism Center (NAC, 2015), and Evidence-Based Practices for Children, Youth, and Young Adults with Autism Spectrum Disorder (Steinbrenner et al., 2020) are developed on the basis of therapies that bring significant improvement to the lives of people with ASD and are recognized as well-established, scientifically verified and recommended for the particular type of difficulties; these are the so-called Evidence-Based Practices (EBP). For ASD, behavioural interventions are best documented, especially in the context of reducing challenging behaviors (Lydon et al., 2017; Watling & Hauer, 2015; Case-Smith et al., 2015). More and more centres and specialists in Poland offer these therapies, yet access to them is still not common (Płatos & Pisula, 2019). Perhaps because of the problem of access to specialized treatment facilities, time consuming procedure and sometimes financial investment in recent years, there has been an increased interest in complementary or alternative therapies, the effectiveness of which are still not convincingly documented, particularly for people with ASD. It has been pointed out that some of these may even have harmful long-term side effects. An interesting review of complementary and alternative therapies in this context is presented by Brondino et al. (2015), including references to the popular Auditory Integration Training and sensory integration in Poland, for which there is still no convincing research or results indicating its usefulness in relation to improving children’s core symptoms of ASD (Sandbank et al., 2020). In spite of these reservations and the lack of satisfactory data regarding the effectiveness of such interventions, the sensory integration therapy is one of the most commonly used forms of therapy for children with ASD in Poland, and is also sought after by the parents (Płatos & Pisula, 2019).

## Problems Related to ASD Diagnostic Process and the Functioning of Specialist Educational and Therapeutic Centres in Poland

Despite our growing knowledge on the subject and improvement of diagnostic procedures, including the development of standardized tools to assist in the diagnosis of autism spectrum disorders, precise guidelines for the diagnostic process itself are still lacking. This means that both the access to and quality of specialist and holistic diagnosis for a child or adult suspected of having ASD vary from one region to another, between facilities or specialists performing the diagnosis. This may result in situations where diagnoses by specialists in the same area, e.g. psychiatrists and psychologists, are mutually exclusive. In their professional practice, the authors sometimes come across situations when the diagnosis put forward by one doctor is rejected by another. This proves burdensome to parents who, considering the welfare of the child, will eventually have to decide which diagnosis they choose as the final one. However, many parents have insufficient knowledge of child development, let alone of autism spectrum disorders. They can hardly be expected to be able to analyse that much information about the specifics of ASD, current diagnostic standards or effectiveness of autism treatment. This leads to a significant increase in parental stress and even more feelings of confusion (Pisula & Noińska, 2011; Pozo et al., 2014; Gosztyła et al., 2020), which is why parents call for the support of specialists who are responsible for accurate diagnosis and treatment plans adapted to the actual demands of the child and the family. For this reason alone, specialists should pay particular attention to the application of the best standards applicable to their discipline in their professional practice. The primary problem in Poland is that of the access to specialized facilities which provide for a comprehensive diagnosis of a child in a situation where autism is suspected, as well as the subsequent therapy, which is an issue already identified several years ago (e.g. Pisula, 2013; Waligórska et al., 2012). In addition to problems of access, waiting times for a diagnosis is still too long, sometimes even a year or more. The costs of obtaining a diagnosis are also high, even though some parents decide to cover the cost of the entire process using private institutions. All other children have to suffer a long waiting time within the framework of state-funded facilities (Pisula, 2013; Płatos & Pisula, 2019). Unfortunately, changes in the approach to autism and attempts to develop better procedures have been associated with the risk of abuse of funds allocated to support people with ASD, for example in educational institutions. In everyday practice, the funds allocated to a child with special educational needs, including autism or Asperger’s syndrome, are not always given to the children who need them most. Since the educational subsidy based on the need for special education is five times higher in the case of children with a diagnosis of autism or Asperger’s syndrome (Ministry of Education Regulation dated 24 August 2017, item 1578 with further amendments) compared to a child without such a diagnosis, some educational facilities take children with ASD who fail to meet most of the diagnostic criteria for the disorder, which means that places for children with autism are often taken by children who do not require such intensive treatment. The 2017 Report of the Supreme Audit Office on the education of children with disabilities (https://www.nik.gov.pl/plik/id,16353,vp,18878.pdf) points to other worrying signals related to providing support to children with ASD. Half of the inspected public schools and nursery schools did not take sufficient care to plan and implement support for children with disabilities, both in the development of individual education and therapy programmes and their implementation by suitably qualified individuals. In the school year of 2016/2017, 24,971 children with autism and Asperger’s syndrome attended Polish schools, which is the largest group after children with mild intellectual disabilities; the figure includes children taught individually within public institutions. Although it must be emphasized that the number of institutions audited was too low to draw clear conclusions from, the results of the Report should provide grounds for initiating a broader discussion on this issue and become an impulse to develop a more uniform model for supporting people with autism spectrum disorders in public educational institutions. The need to develop and improve the quality of support provided to children with autism spectrum disorder at school is also connected to the fact that there are significant inequalities in the provision of services for children with ASD at national level. For children from small towns and villages, whose access to specialist centres is limited due to their remote location, the school and the rehabilitation it provides are the only forms of therapeutic support they receive. Deeper reflection is also needed in relation to the issue of adults with autism, as specified by the report Autism-the situation of adults (Jankowska et al., 2014). Even though the Polish Education system provides a quite extensive support system for children with ASD, it is drastically reduced for school leavers. In fact no statistics are available on the number of adults with autism at the municipality level, but we know there are too few daytime centres for adults, adjusted to the difficulties they experience. The issue has been recently often discussed and analysed also at the public administration level (e.g.. https://www.nik.gov.pl/plik/id,22196,vp,24863.pdf). A discussion has taken place recently among the circles linked to support provision to people with ASD and the state authorities with regards to the disability assessment, particularly of children with Asperger Syndrome. What gave grounds for this debate was the reservations put forward by the Ombudsman for People with Disabilities as to the accuracy of specialist diagnosis and final assessments, which result in overrepresentation of children with Asperger Syndrome in the education system. The Ombudsman’s doubts regarded the issue of how the relevant educational subsidy is spent. As it is, the benefits children with ASD receive is the same as the children with Asperger Syndrome. However, according to the government representative, the level of support required by pupils with childhood autism is higher than by children with Asperger Syndrome. According to the representative of the circles supporting people with ASD, this showed insufficient preparation and knowledge of the government representatives, which led to unnecessary anxiety not only among parents and people with ASD but also the specialists who support them. It has also showed some of the shortcomings of the Polish disability assessment system and pointed towards the need to develop new systemic solutions (see: http://psych.uw.edu.pl/wpcontent/uploads/sites/98/2021/01/Spotkanie_z_Min_Pawlem_Wdowikiem_podsumowanie_2_02_2021.pdf).

## Summary and Conclusions

As the etiology of ASD is still not fully understood, standardized and unified diagnostic procedures are lacking and new challenges for specialists are continually emerging, the issue of autism is thought to involve many complex, difficult-to-solve problems in the settings in which decisions are often saturated with a significant risk of error. It is particularly important that the situation does not lead to negligence and mistakes in decision-making, which would have serious negative consequences for ASD sufferers, their families and the society at large. It is therefore important that ethical considerations are taken into account at every stage of formulating and implementing programmes to support these people. In view of the phenomena observed in the area of the provision of support to children and adults with ASD, it seems necessary to introduce an effective, professional system of monitoring the services provided, possibly in the form of certification and control, in order to prevent their abuse.

Perhaps it would even make sense to introduce the category of temporary diagnosis, in this way admitting to the uncertainty of the diagnostic process, which would still in practical terms give individuals access to specialist therapy and the benefits of institutional support. This would mean that the responsibility for the diagnostic uncertainty is shifted to specialists and it would give the family time to focus on the child’s health, and consequently avoid the trauma associated with an ASD diagnosis (Casey, 2012).

There is also a need to rationalize concepts and areas of diagnostic uncertainty or ambiguity, to disseminate or develop diagnostic techniques to provide insight into adequate categorizations with which to differentiate ASD symptoms from other disorders, personality traits regarded as the norm, features of personality disorders, family contexts, traumatic experiences, etc. In order to avoid the ASD—non-ASD dichotomy, we should refer the child’s behaviour to the varied terminology that covers different areas of the norm and correct differentiation of the disorder. Too many children get similar diagnoses. The increased frequency of ASD, ADHD and SI diagnoses in Poland in recent years has raised doubts about their accuracy and their misuse by doctors or unauthorised professionals (psychologists, pedagogue, teachers) (Borkowska & Wagh, 2010, pp.7–10; Gawęda et al., 2010; Kruk-Lasocka, 2012; Ochojska & Pasternak, 2020; Szaniawska, 2010). The role of multi-specialist, comprehensive and possibly most objective diagnosis should be emphasized. It is also worth remembering that the global trend of growing numbers of diagnosed disorders comorbid with ASD is not sufficiently practised in Poland.

The subjective weight of an ASD diagnosis is considerable. Both its underestimation and overestimation have very serious consequences, in fact more serious than is the case with many other disorders. Based on the publications available in Poland, the content of ASD conferences and the analysis of children’s assessments presented at psychological-pedagogical counseling centers and mental health clinics, it appears that the diagnosis of ASD in Poland relies too much on the perspective of the external observer: the symptoms observed are assigned to appropriate categories, forgetting other important elements of the diagnosis, i.e. the search for alternative explanations and the internal perspective. Teachers and specialists frequently say of a child’s problems: “she does not look others in the eye”, “he is spinning around aimlessly”, or “he plays with the same toy excavator all the time". It is worthwhile for diagnosticians to think about discovering the child’s inner perspective (even in the form of a working hypothesis) and then decide whether it can be cross-referred to the norm, ASD or another disorder. In fact these externalized statements need to be reformulated to internal-subjective ones; perhaps instead of ‘he is spinning around aimlessly’ we might say ‘he does not like for the play to be interrupted, neither does he like tidying up. He misses parents and is coping with the tension in this way without any other good solution presenting itself to him. It is also important to consider the adaptation problems that a child might have, especially during the initial period at a pre-school or school (Ochojska & Pasternak, 2020). We suggest that a good diagnosis is created by combining a few perspectives of the child’s situation: (a) a viewpoint related to general condition of the child’s health; (b) nosological, psychometric and functional perspective; (c) systemic and social perspective, making a real attempt to understand symptoms and underlying calming and exacerbating factors in the context of family and social setting; (d) emphasizing resources and other areas related to health. Focusing much attention on the problems of autism may have the effect of growing number of suspected ASD cases and therefore of its diagnosis. The pattern can be summarized as follows: a wide-ranging information and training on ASD becomes available, focusing mainly on the inclusion criteria rather than the differential diagnosis involving the knowledge of other disorders. As a result, the nursery or primary education professionals pay inadequately high attention to identifying children displaying the symptoms they have learnt about and suggest a visit to a neurologist. The neurologist considers the teacher’s assessment and issues a fairly ambiguous certificate suggesting ‘possible ASD’ or the disorder with the features of autism. The family then takes the certificate to a psychiatrist or to the diagnostic centre and, at this point, it can go either way. If the diagnostician considers the previous information about the child while the diagnosis itself is inconclusive, it can work in favour of the ASD diagnosis and, in this way, the child and the family acquires the new identity of ‘having a disorder”. The referral institution rests assured that they were right in the first place and gets the funding for the therapy of the child with autism. The community of people involved in the diagnosis, treatment, conducting research and dissemination of its results is undoubtedly committed to raising the public awareness of ASD through training and conferences. The participants in such events (e.g. pre-school, school teachers, educators, psychologists) acquire knowledge and learn practical skills but it is also important that the training and conference organizing circles made sure of the legal boundaries and the scope of this knowledge. After all, it is often the case that such “educated” persons exceed the range of their competences or responsibilities and take a position, either formally or informally, diagnosing ASD in people around them.

This might have contributed to the development of a kind of “diagnostic and therapeutic industry”, or at least in some cases pre-schools hire or asks a diagnostician to identify children “with ASD characteristics” and then direct them to this specialist for further diagnosis, where the suspicion is confirmed. Then the child with confirmed ASD goes back to the school, which employs an additional specialist, the child attends the facility “for free”, whereas in fact he or she benefits from high educational subsidies, while the original diagnostician or someone working directly with them becomes responsible for the therapy. In the professional experience of one of the authors, many children who are referred for confirmation of diagnosis fail to meet the ASD diagnostic criteria or make a significant progress in speech therapy. Similar diagnositc difficulties have been made by Kruk-Lasocka (2012). This is why it is so important to ensure the independence of the diagnosis.

Unfortunately, there is an insufficient availability of diagnostic and therapeutic facilities which provide for developing interests and social networking opportunities for teenagers and adults with ASD (Płatos et al., 2016). Also few professionals are expert or can train others who can help people with ASD deal with their sexuality (Fornalik, 2020). More attention should be also paid to potential suicide attempts by people with ASD, to enable them to benefit from psychological and psychiatric support in a presuicidal crisis and in the period after suicide attempts.

The good point and irregularities mentioned in this article refer to both diagnostic and therapeutic issues. It seems that there is a strong need for developing the correct areas of diagnosis (and therapy) in Poland, to develop diagnostic standards, unifying procedures and ensuring a thorough training for diagnosticians. The lack of such a system may lead to various abuses in this area exposing the system to mismanagement of funds.
